# Mandatory oral glucose tolerance tests identify more diabetics in stable patients with chronic heart failure: a prospective observational study

**DOI:** 10.1186/1758-5996-6-44

**Published:** 2014-03-27

**Authors:** An LM Stevens, Dominique Hansen, Vincent Vandoren, Rob Westerlaken, An Creemers, Bert O Eijnde, Paul Dendale

**Affiliations:** 1 REVAL Rehabilitation Research Centre, Hasselt University, Martelarenlaan 42, BE-3500 Hasselt, Belgium; 2 Heart Centre Hasselt, Jessa Hospital, Stadsomvaart 11, BE-3500 Hasselt, Belgium; 3 I-BioStat, Hasselt University, Martelarenlaan 42, BE-3500 Hasselt, Belgium

**Keywords:** Insulin resistance, Body composition, Muscle strength, Quality of life

## Abstract

**Background:**

Many patients with chronic heart failure (CHF) are believed to have unrecognized diabetes, which is associated with a worse prognosis. This study aimed to describe glucose tolerance in a general stable CHF population and to identify determinants of glucose tolerance focusing on body composition and skeletal muscle strength.

**Methods:**

A prospective observational study was set up. Inclusion criteria were diagnosis of CHF, stable condition and absence of glucose-lowering medication. Patients underwent a 2 h oral glucose tolerance test (OGTT), isometric strength testing of the upper leg and dual energy x-ray absorptiometry. Health-related quality of life and physical activity level were assessed by questionnaire.

**Results:**

Data of 56 participants were analyzed. Despite near-normal fasting glucose values, 55% was classified as prediabetic, 14% as diabetic, and 20% as normal glucose tolerant. Of all newly diagnosed diabetic patients, 79% were diagnosed because of 2 h glucose values only and none because of HbA1c. Univariate mixed model analysis revealed ischaemic aetiology, daily physical activity, E/E’, fat trunk/fat limbs and extension strength as possible explanatory variables for the glucose curve during the glucose tolerance test. When combined in one model, only fat trunk/fat limbs and E/E’ remained significant predictors. Furthermore, fasting insulin was correlated with fat mass/height^2^ (r = 0.51, p < 0.0001), extension strength (r = -0.33, p < 0.01) and triglycerides (r = 0.39, p < 0.01).

**Conclusions:**

Our data confirm that a large majority of CHF patients have impaired glucose tolerance. This glucose intolerance is related to fat distribution and left ventricular end-diastolic pressure.

## Background

Chronic heart failure (CHF) is a system disease. Apart from cardiac failure, the clinical picture involves pulmonary, renal, hepatic and skeletal muscle abnormalities [1]. In addition, diabetes mellitus type 2 is frequently found, with percentages varying from 8-41% [2]. Although the suspicion arises that impaired glucose tolerance is the rule rather than the exception in this population, its exact prevalence is not known [2,3].

In CHF patients with reduced systolic function, left ventricular ejection fraction and aetiology of CHF have been described as predictive factors for insulin sensitivity [4-9]. Furthermore, typical CHF medical therapies, i.e. ACE inhibitors , β-blockers and thiazides are believed to influence glucose tolerance [10-14].

Although a higher body mass index is associated with impaired glucose tolerance and diabetes, it is also associated with better survival in CHF [15]. When dividing body weight into fat mass and lean mass, it could be hypothesized that a higher fat mass leads to overall detrimental effects, while a higher lean mass is associated with reduced catabolism and beneficial effects in CHF [16]. In addition, a higher muscle mass could lead to elevated glucose uptake. Precise measurement of body composition would therefore provide new insights in the relation between body composition and glucose tolerance in CHF.

As skeletal muscle strength is also an independent predictor for survival in CHF, it could be hypothesized that not only the quantity but also the quality of skeletal muscle plays an important role [17]. Still, skeletal muscle function has not been investigated in relation to glucose tolerance in CHF yet.

Following this line of reasoning, the present study aims to describe glucose tolerance in relation to (a) severity of heart failure, (b) the intake and dosage of typical CHF medical therapies and (c) body composition and skeletal muscle strength in a heterogeneous group of stable CHF patients.

## Methods

### Subjects

Patients diagnosed with CHF were recruited from the heart failure clinic of the Jessa hospital (Hasselt, Belgium). Inclusion criteria were (1) a history of CHF of at least 6 months and (2) clinically stable for more than 3 months prior to the onset of the study. Known diabetes with glucose lowering therapy, engagement in phase III rehabilitation in a hospital setting and other chronic diseases (pulmonary disease, end-stage renal disease, cancer) were the exclusion criteria. Based on a previous study, sample size was estimated on 60 patients [7]. All patients gave their written informed consent. Ethical approval of the study was obtained from the committees of the Jessa hospital and Hasselt University. The investigation conforms with the principles outlined in the Declaration of Helsinki.

### Study design

In this prospective observational study, patients underwent a 2 h oral glucose tolerance test (OGTT), had a late breakfast and muscle strength and body composition were assessed on a single test day. During the OGTT, health-related quality of life and physical activity level questionnaires were completed and current medical therapy was registered.

### Oral glucose tolerance test and blood parameters

Following an overnight fasting period, baseline blood glucose and insulin concentrations as well as blood lipids, HbA1c and B-type Natriuretic Peptide (BNP) were determined via a venous blood sample. Hereafter, 75 g glucose (Merck KGaA, Darmstadt, Germany) dissolved in 250 mL water was ingested and 1- and 2 hours blood samples were taken for glucose and insulin analysis. Blood samples for glucose and insulin (in serum separation tubes) and BNP (in EDTA tubes) were maintained at room temperature for 30 min, centrifuged, and the collected serum and plasma were frozen at -80°C until analysis. Blood samples for lipids (in lithium heparin tubes) and HbA1c (in EDTA tubes) were processed on the test day. Glucose, total cholesterol and HDL cholesterol were determined with an Olympus AU analyzer (Beckman Coulter, Switzerland), insulin and BNP with ADVIA Centaur (Siemens Medical Solutions Diagnostics, Germany) and HbA1c with Hi- Auto A1C Analyzer (Menarini Diagnostics, Italy). Serum glucose was converted to plasma glucose using the following formula: plasma glucose (mmol/L) = -0.137 + (1.047* serum glucose (mmol/L)) [18]. Subjects were divided into 3 groups according to their glucometabolic state as recommended by the American Diabetes Association (see Table 1) [19]. Reference values for insulin during OGTT were 3-28 mU/L at fasting state, 29-88 mU/L 1 h after glucose load and 22-79 mU/L 2 h after glucose load [20].

**Table 1:** Criteria used for glucometabolic classification

	**NGT**	**Prediabetes**	**Diabetes**
Fasting glucose	<5.6 mmol/L	5.6-6.9 mmol/L	≥7.0 mmol/L
OR			
2-hour glucose	<7.8 mmol/L	7.8-11.0 mmol/L	≥11.1 mmol/L
OR			
HbA1c	<5.7%	5.7-6.4%	≥6.5%

*NGT*, normal glucose tolerance.

### Muscle strength

Maximal voluntary unilateral strength of the upper leg was evaluated in a seated position on an isokinetic dynamometer (System 3; Biodex Medical Systems, New York, USA). The rotational axis of the dynamometer was aligned with the transverse knee joint axis and connected to the distal end of tibia. Subjects performed 2 maximal isometric knee extensions and flexions at knee angles of 45° and 90°. Maximal contractions (4 s) were interspersed by 30 s rest intervals. The highest isometric extension and flexion torques (Nm) at each knee angle were selected as peak torque. Maximal strength was expressed as peak torque relative to lean tissue of the right leg.

### Body composition

To determine body composition, a Dual Energy X-ray Absorptiometry scan (Hologic, Vilvoorde, Belgium) was performed. Fat tissue mass and lean tissue mass were obtained for the whole body and for the following separate regions: legs, trunk, gynoid and android region. From these findings, the following indices were calculated: waist-to-hip fat mass ratio (android fat (g)/gynoid fat (g) ratio), fat of the trunk/fat mass of the limbs ratio.

### Medical history and echocardiography

Hospital records were retrospectively reviewed for aetiology of heart failure (ischaemic versus non-ischaemic), and for left ventricular ejection fraction (EF) in the most recent echocardiography. In 25 patients, echocardiography was performed in the month following OGTT, with determination of E/E’.

### Health related quality of life and physical activity

The EQ-5D was used to evaluate health related quality of life. It is a standardized, non-disease-specific instrument for describing and valuing health, which is limited in length (5 short questions and a visual analogue scale) [21,22]. Daily physical activity was assessed using the International Physical Activity Questionnaire.

### Statistical analysis

Statistical analyses were performed using SAS Enterprise Guide 4.3, SAS 9.2 (SAS Institute Inc., Cary, NC) and R2.10.1 software. All measures are presented as mean ± SD. Continuous data were compared using nonparametric one way ANOVA and *post hoc* multiple comparison procedures were performed using Wilcoxon rank sum test with Bonferroni correction. Categorical data were compared using Fisher exact test. Because the glucose curve consists of longitudinal data (3 time points for each patient), relations between the glucose curve and other patient characteristics (explanatory variables) were investigated with mixed model analysis. Multiple imputation (number of imputed datasets = 5) was performed for E/E’ based on the glucose levels during OGTT [23]. Bivariate correlation (Spearman) was performed between fasting insulin and predictive variables. All tests were two-sided with a P-value of 0.05 as threshold for statistical significance.

## Results

**Figure 1:**
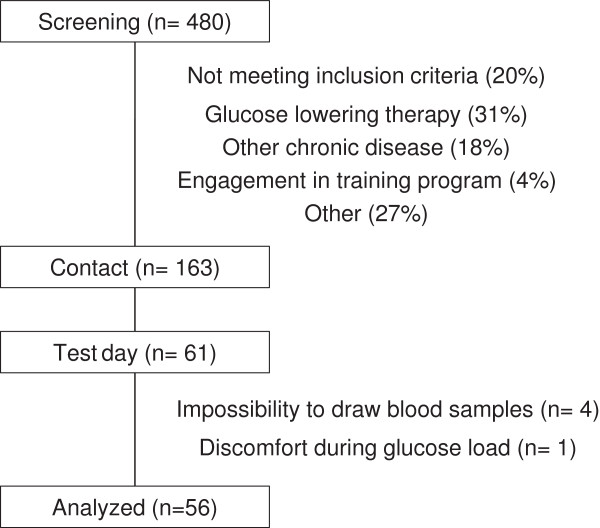
Patient flow diagram.

From March 2011 to March 2012, a total of 480 patients were screened. A patient flow diagram is presented in Figure 1.

### Oral glucose tolerance test

**Figure 2:**
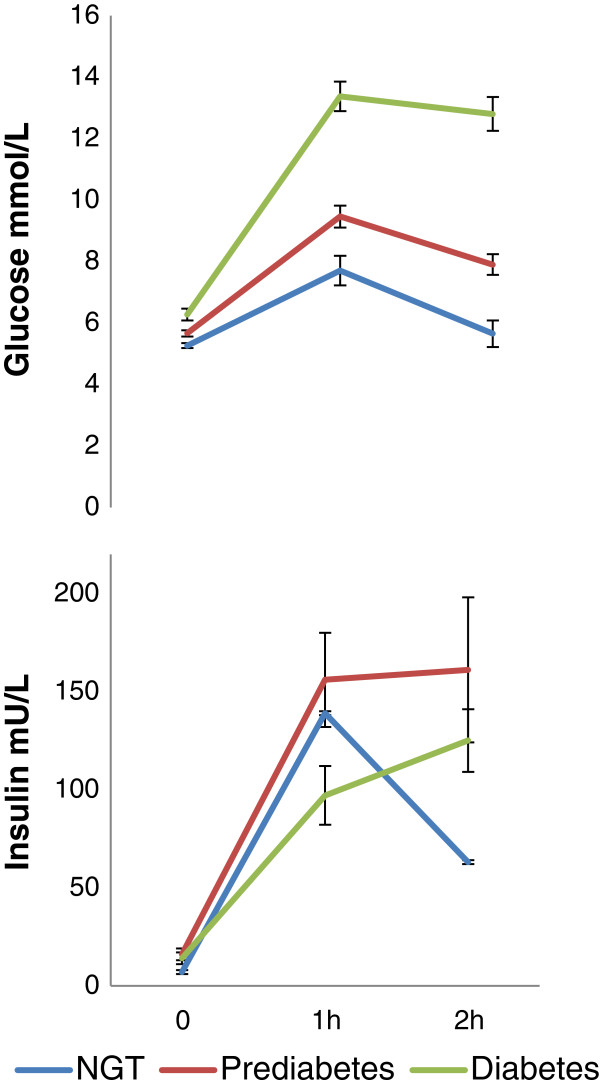
**Glucose and insulin concentrations during 2 h OGTT.** Data are shown as mean ± SE. NGT: normal glucose tolerance.

Despite near-normal mean fasting glucose in the total group (5.7 ± 0.6 mmol/L), 14 patients (25%) were classified as having newly diagnosed diabetes, 31 (55%) as having prediabetes and 11 (20%) as having NGT. Overt diabetes was diagnosed because of 2 h glucose values only in 79% of patients, while only 3 patients could be diagnosed because of fasting glucose values and none because of HbA1c (Table 2). Glucose and insulin curves during OGTT are presented in Figure 2. In all 3 groups, mean fasting insulin levels were in the normal range but increased above reference values 1 h after glucose load. Two hours after glucose load, insulin curves did not decline in prediabetics and diabetics.

**Table 2:** Importance of 2-hour glucose values for glucometabolic classification

	**Prediabetes n = 31**	**Diabetes n = 14**
Fasting glucose	5 (16%)	1 (7%)
Fasting + 2 h glucose	4 (13%)	2 (14%)
Fasting glucose + Hba1c	6 (19%)	-
Fasting glucose + 2 h glucose + HbA1c	3 (10%)	-
2-hour glucose	4 (13%)	11 (79%)
2 h glucose + HbA1c	6 (19%)	-
HbA1c	3 (10%)	-

Data are presented as frequency and percentage.

### Patient characteristics and body composition

Table 3 shows patient characteristics of the 3 groups. Differences in New York Heart Association class distribution and BNP values between groups were not significant. Medical treatment, EF, age, gender, whole body weight and fat mass were comparable between groups. Fat distribution expressed as fat trunk/fat limb ratio tended to be higher in diabetics compared to prediabetics and NGT (p < 0.03). However, these differences did not reach significance after Bonferroni correction.

**Table 3:** Comparison of patients characteristics according to glucometabolic state

	**NGT n = 11**	**Prediabetes n = 31**	**Diabetes n = 14**	**p**
Age (years)	64 ± 17	69 ± 11	70 ± 11	.58
Sex (% male)	91	58	64	.15
BMI (kg/m^2^)	25.1 ± 3.7	27.6 ± 5.5	29.9 ± 9.7	.39
Aetiology (% IHD)	27	32	57	.22
NYHA class (% I-II-III)	55-36-9	23-48-29	21-57-21	.34
LVEF (%)	43 ± 13	42 ± 12	40 ± 12	.71
E/E’ (n = 5, n = 13, n = 7)	21.4 ± 19.4	21.2 ± 11.0	15.0 ± 8.7	.33
ACE-inhibitor or AIIA (%)	100	74	86	.16
% optimal daily dosage	73 ± 26	96 ± 32	88 ± 61	.15
Selective β-blocker (%)	73	77	86	.75
% optimal daily dosage	41 ± 17	52 ± 27	52 ± 25	.56
Non-selective β -blocker (%)	18	13	7	.75
% optimal daily dosage	150 ± 71	137 ± 75	50 ± 0	.40
Diuretic (%)	64	84	71	.32
% usual daily dosage	113 ± 67	103 ± 73	129 ± 106	.75
BNP (pg/mL)	167 ± 120	177 ± 207	293 ± 383	.39
HbA1c (%)	5.3 ± 0.2	5.7 ± 0.3	5.9 ± 0.3	.01^*^
Total cholesterol (mg/dL)	176 ± 39	173 ± 45	169 ± 53	.81
HDL cholesterol (mg/dL)	43 ± 11	50 ± 15	49 ± 15	.39
LDL cholesterol (mg/dL)	107 ± 29	93 ± 32	88 ± 42	.25
Triglycerides (mg/dL)	131 ± 35	159 ± 101	160 ± 88	.94
Body weight (kg)	76.5 ± 15.8	76.5 ± 16.5	82.9 ± 30.0	.10
Fat mass (%)	31.1 ± 6.9	35.2 ± 9.1	34.8 ± 9.2	.52
Lean mass (kg)	52.2 ± 9.3	49.3 ± 9.7	52.2 ± 12.4	.62
Fat trunk/fat limb ratio	1.28 ± 0.39	1.39 ± 0.26	1.63 ± 0.35	.03**
Extension strength (Nm/kg)				
45°	16.8 ± 6.1	13.9 ± 2.9	13.7 ± 5.1	.32
90°	16.6 ± 3.0	15.4 ± 4.1	15.3 ± 6.4	.58
Flexion strength (Nm/kg)				
45°	9.0 ± 2.8	8.2 ± 2.2	7.8 ± 3.4	.89
90°	7.5 ± 2.2	6.7 ± 1.9	6.2 ± 2.4	.60
Self-reported physical activity (METminutes/day)	1921 (533-4313)	1872 (1789-4047)	1219 (664-5686)	.73

*BMI*, body mass index; *BNP*, B-type natriuretic peptide; *IHD*, ischaemic heart disease; *LVEF*, left ventricular ejection fraction; *NGT*, normal glucose tolerance; *NYHA*, New York heart association functional class. Extension and flexion strength are expressed as peak torque relative to lean tissue of the right leg. Continuous variables are presented as mean ± SD, physical activity is presented as mean and 95% confidence interval. Categorical variables are presented as number and percentage.

*NGT versus prediabetes and diabetes, **post hoc differences not significant after Bonferroni correction.

### Muscle strength

Maximal isometric strength was comparable between groups.

### Health related quality of life and physical activity

The total score of the EQ5D was similar in all 3 groups (data not shown), as well as overall self-rated health status (NGT: 7.0 ± 1.0, prediabetics: 6.7 ± 1.9, diabetics: 6.4 ± 2.0; p = 0.90). Also reported daily physical activity did not differ between groups.

### Contributors to glucose tolerance

Univariate mixed model analysis revealed 6 possible explanatory variables for the glucose values during OGTT: ischaemic aetiology, daily physical activity, E/E’, android/gynoid fat ratio, fat trunk/fat limbs and knee extension strength at 90°. Android/gynoid fat ratio was excluded from the final model building because of interference with the other fat distribution variable. When the remaining 5 variables were combined in one model with random intercept, and after manual backward selection procedure, fat distribution showed a main effect for overall glucose values. Furthermore, E/E’ and fat distribution also showed an interaction effect with glucose curve over time. Multiple imputation analysis produced the same results.

Because insulin release after glucose loading was decreased in the diabetic group, and the insulin curves therefore did not follow the same increasing trend as the glucose values, mixed model analysis was not performed for the insulin curves. Instead, fasting insulin was found to correlate moderately with body mass index (r = 0.51, p < 0.0001) and fat mass/height^2^ (r = 0.51, p < 0.0001). Fasting insulin was also slightly related with total body fat (%; r = 0.49, p = 0.0001), triglycerides (r = 0.39, p < 0.01) and quadriceps strength at 45° (r = - 0.33, p = 0.01).

## Discussion

Despite near-normal fasting glucose values, our data show that the majority of stable CHF patients have impaired glucose tolerance. This was related to body composition and left ventricular end-diastolic pressure, but not to severity of heart failure symptoms, severity of left ventricular dysfunction, nor typical medical therapies.

### Description of glucose tolerance

In our study population, 80% showed an abnormal reaction to glucose intake. This was easily detected using a 2 h oral glucose tolerance test, as proposed by the American Diabetes Association [19]. In fact, only 21% of patients with diabetes would be correctly classified using fasting glucose values only, and none using HbA1c.

Glucose values were in the prediabetic range in 55% of the patients, and in the diabetic range in 25%. The proportion of prediabetes and undetected diabetes is much higher in this study when compared to the findings of other studies, reporting 22-23% prediabetes and 18% newly diagnosed diabetes in a selected population of CHF patients with reduced EF [3,9]. This may be due to the heterogeneous study population irrespective of EF. Furthermore, it is related to the use of stricter glucose cutoff values in addition to HbA1c values for diagnosis of (pre)diabetes in the present study.

The prognostic impact of this finding is enormous. A stepwise increasing mortality rate with increasing glucose intolerance assessed by OGTT was found by Egstrup et al. [24]. Consequently, 80% of the study population, which represented the general CHF population without glucose lowering therapy in the heart failure clinic, is at higher risk for mortality compared to other CHF patients with normal glucose tolerance. Therefore, the transition into overt diabetes and worse prognosis should be prevented. Treatment of (pre)diabetic CHF patients with glucose lowering medication is not evident, as they may be contra-indicated in this population [25]. Therefore, diet counseling and exercise therapy are the preferred treatment methods. Although evidence points to a possible beneficial effect of exercise therapy on whole body glucose uptake in CHF, results are not conclusive. More studies using standardized glucose tolerance assessment and supervised exercise interventions are needed [26].

### Glucose tolerance in relation to severity of heart failure

Previous studies describing EF as a determinant for glucose tolerance were performed in CHF patients with reduced EF [4,7,27]. In addition, an association between diastolic function and impaired glucose tolerance in CHF patients with preserved EF has been reported [28-30]. Because we included patients with reduced as well as preserved EF, we expected to find a relation between glucose tolerance and EF with worse glucose tolerance in patients with preserved EF. However, mixed model analysis showed no influence of EF on overall glucose values or shape of the glucose curve. Likewise, BNP was not associated with glucose curve. Also, a stepwise increase along the diabetic continuum as shown by Stahrenbergh *et al* and Dinh *et al* was not present in this study population [28,30]. On the other side, E/E’, a marker of left ventricular end-diastolic pressure, appeared to be a contributing factor for glucose response during OGTT.

### Glucose tolerance in relation to typical CHF medical therapies

Almost all patients in the study group were optimally treated, and therefore pharmacological treatment between groups was similar. Our hypothesis that glucose tolerance is related to the intake and dosage of typical CHF medical therapies was therefore not confirmed.

### Glucose tolerance in relation to body composition and skeletal muscle strength

The classic link of glucose intolerance with increasing obesity was confirmed. Interestingly, a greater importance of fat mass and fat distribution (trunk fat/limb fat) was shown compared to lean mass. The ideal body weight for CHF patients has been the subject of debate. On the one hand, a low body mass index is a risk factor for mortality in CHF while the presence of obesity (body mass index 30-35 kg/m^2^) is associated with lower mortality [15]. On the other hand, more detailed body composition parameters may give another view on the beneficial effects of obesity in CHF. Oreopoulos *et al* suggested that higher lean mass is protective in CHF, while fat mass is associated with detrimental effects as higher fasting glucose [16]. As higher fat mass and fat distribution around the trunk were predictors for glucose response in the present study, our data agree with this logic. However, although lean mass was quantitatively not different between groups, it is probable that muscle quality and function are decisive factors for glucose tolerance. In this respect, Doehner et al have showed the decreased glucose transporter protein type 4 in skeletal muscle of CHF patients, independent of body composition [5].

Muscle function in terms of extension strength of the upper leg was related to overall glucose values, but was not a predictor for glucose tolerance when combined with other variables. In addition, extension strength was negatively correlated with fasting insulin values. This confirms our hypothesis that higher skeletal muscle strength is associated with better glucose tolerance. Although we believe that higher muscle strength is a reflection of increased levels of physical activity, this was not confirmed by the results of physical activity assessment with the International Physical Activity Questionnaire.

This study has some limitations. Echocardiographic data were retrieved from hospital records and were not prospectively assessed. Physical activity was assessed by questionnaire, although it does not reflect true physical activity as compared to pedometers and accelerometers. Furthermore, cardiopulmonary exercise tolerance and isokinetic strength endurance assessment could have added valuable information to the predictive model. Finally, the study did not include healthy controls.

## Conclusions

The proportion of glucose intolerant CHF patients is alarmingly high, and is underestimated when screening only fasting glucose and HbA1c. Our data did not show an association between glucose tolerance and EF, New York Heart Association class, nor medication use. However, glucose tolerance was associated with left ventricular end-diastolic pressure and body fat distribution.

## Abbreviations

CHF: Chronic heart failure; EF: Ejection fraction; OGTT: Oral glucose tolerance test.

## Competing interests

The authors declare that they have no competing interests.

## Authors’ contributions

AS conceived of the study, performed the data collection, statistical analysis, interpretation of data and drafted the manuscript. DH participated in interpretation of data and drafting the manuscript. VV and RW helped setting up the study, performed data collection and statistical analyses. AC gave statistical counseling and performed statistical analyses (mixed models). BO and PD were involved in the conceiving of the study and in the interpretation of data as well as drafting the manuscript. All authors read and approved the final manuscript.
